# The ‘Red Herring’ Hypothesis: Some Theory and New Evidence

**DOI:** 10.3390/healthcare10020211

**Published:** 2022-01-21

**Authors:** Peter Zweifel

**Affiliations:** Department of Economics, University of Zurich, Rämistr. 71, 8006 Zürich, Switzerland; peter.zweifel@uzh.ch

**Keywords:** ‘red herring’ hypothesis, gender difference in healthcare expenditure, age profile of healthcare expenditure, time to death, rationing

## Abstract

The ‘red herring’ hypothesis (RHH) claims that apart from income and medical technology, proximity to death rather than age constitutes the main determinant of healthcare expenditure (HCE). This paper seeks to underpin the RHH with some theory to derive new predictions also for a rationed setting, and to test them against published empirical evidence. One set comprising ten predictions uses women’s longer life expectancy as an indicator of the difference in time to death in their favor. Out of 28 testing opportunities drawn from the published evidence, in the case of no rationing seven out of eleven result in full and two in partial confirmation; in the case of rationing, twelve out of 17 result in full and one in partial confirmation. The other set, containing 35 testing opportunities, concerns the age profile of HCE. In the case of no rationing, seven out of twelve result in full and four in partial confirmation; in the case of rationing, eleven out of 23 in full and nine in partial confirmation. There are but ten contradictions in total. Overall, the new tests of the RHH can be said to receive a good deal of empirical support, both from countries and settings with and without rationing.

## 1. Introduction

According to conventional wisdom, age and sex are the crucial determinants of a person’s healthcare expenditure (HCE); accordingly, the future aging of population is predicted to cause a continuing surge in HCE. This view was challenged by [1] who arguably were the first to be able to distinguish three concepts of time, viz. (i) historical time reflecting medical technology, (ii) calendar age, and (iii) time to death. This was possible thanks to a panel dataset which recorded also the insured’s time of death. Since, in a regression, concepts (i) and (iii) proved significant but not concept (ii), the authors concluded that the focus on age and aging was a ‘red herring’, detracting attention from new medical technology as the driver of HCE. This ‘red herring’ hypothesis (RHH henceforth) has been subject to a debate that is not resolved to this day. It states that (apart from income and medical technology) proximity to death rather than age is the main driver of healthcare expenditure (HCE). There are several variants of the RHH hypothesis. While [2] distinguish no fewer than four versions, the one adopted here is their no. 3, “In a regression equation for individual HCE, the age variable(s) become(s) weak or zero, once time-to-death (TTD) is included”). Of course it is true that with each year, all individuals are closer to death *ceteris paribus*, the process of population aging does not imply increasing proximity to death. To the contrary, in the future persons aged 80 (say) may well be farther away from death and hence the costly final years of life than at present—if the RHH is true. Given constant medical technology, future cohorts may even cause less HCE on a lifetime basis because the years with high HCE account for a lower share of their total lifespan.

**Figure 1:**
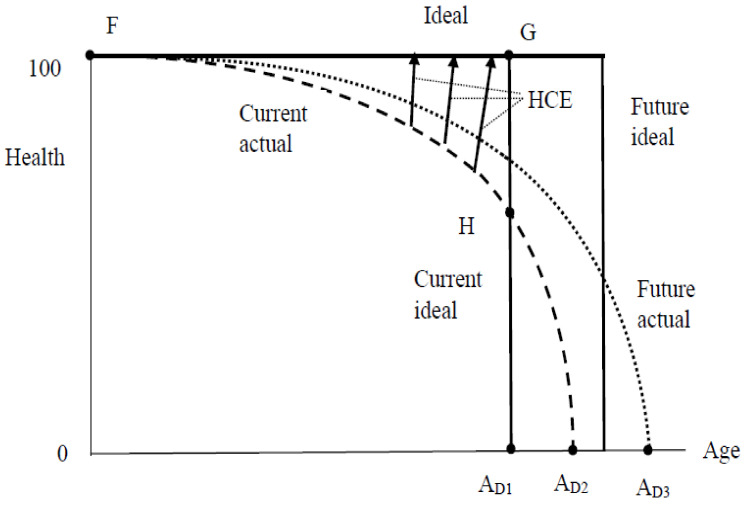
Ideal and actual health profiles, and HCE.

Interestingly, a theoretical justification of the RHH seems to be lacking. Figure 1 below is designed to fill this gap in a simple way. The solid rectangular graph reflects the desire to be 100 percent healthy until the time has come to drop dead (indicated by A_D1_), an ideal shared by most western (or westernized) cultures [3,4]. The actual age profile follows the dashed line with age at death indicated by A_D2_. For the two profiles shown, second-degree stochastic dominance (SDSD) predicts that people prefer the ideal profile to the actual one in spite of later death since the area FGH exceeds the area HA_D1_A_D2_ (When Figure 1 is turned around to make the Health axis the horizontal one, it becomes evident that the two health profiles can be interpreted as cumulative distribution functions, indicating the probability mass of “Health” up to a given age. This justifies application of the SDSD criterion). This implies that people are willing to make effort to return to the ideal rectangular age profile, importantly in the guise of HCE. However, with increasing closeness to death, the greater becomes the difference between actual and desired health status, triggering an increasing amount of HCE. Note that this argument holds regardless of age; to see this, consider a (likely) future where age at death increases to A_D3_, along with an extension of the ideal age profile of health. Once again, HCE (arrows originating from the line FA_D3_ not shown) increases with closeness to death.

Admittedly, this line of argument is somewhat simplistic because with increasing age, the likelihood of being able to return to the ideal profile decreases, possibly causing individuals to “pull the plug” and to let HCE drop to zero (see [5,6] for a pertinent theoretical development).

Yet if (approximately) true, the RHH has an important implication for policy. The conventional wisdom is that the aging of a population will cause a cost explosion in health care, most recently in the context of long-term care [7]. However, provided deviations from the ideal health profile do not start earlier in life [for which there is no evidence, to the contrary; see [8]), HCE (and likely long-term care expenditure) will continue to be concentrated on a few years prior to death. This leaves the pace of technological change in medicine as the crucial determinant of future HCE at the individual level.

In spite of the intuitive justification provided above, the RHH has not been fully confirmed by empirical evidence. [9] even proclaimed “the death of the red herring”, based on their finding that age continues to be a significant determinant of HCE even when time to death is included in the regression equation. However, a review of the literature (see Table A1 of Appendix B) reveals that the majority of the contradictory findings originate from countries and settings subject to rationing (as is true also of [9]). In these cases, observed HCE must not be interpreted as the outcome of patient demand only; rather, it reflects the influence of physicians as rationing agents, resulting in a demand- and supply-side interaction to be modeled in Section 2.1 below. 

Section 2.2 of this paper groups countries and settings according to whether they are characterized by rationing of health care or its absence, respectively. This is important for the development of two sets of new predictions derived from the RHH. The first set, presented and tested in Section 3.1, uses women’s higher remaining life expectancy (RLE) as an indicator of time to death. In OECD countries, females have a longer RLE than males both at birth and at age 65, suggesting that this difference holds across all ages [10]. Causes of this difference include biological factors favoring women (hormones, metabolism), social factors (work stress, networks), and behavioral factors (risk taking, alcohol and substance abuse) [11]. 

In the present context, women’s longer RLE as a determinant of a difference in HCE needs to be qualified by their higher willingness to pay (WTP) for health care, which is an important driver of HCE in the absence of rationing as soon as patients have to pay for medical care out of pocket. There is evidence suggesting that women have higher WTP than men even when controlling for income. [12] obtained a higher estimated WTP for the prevention of influenza among female compared to male employees if beneficiaries were other employees in the vaccinated subgroup (with the difference lacking statistical significance in the non-vaccinated one) and if beneficiaries are other adult household members in the non-vaccinated subgroup. While gender-specific estimates of WTP for the same type of medical care are rare, [13] do find it to be significantly higher for Japanese women in the case of myocardial infarction (but only insignificantly so in the case of the common cold and retinal detachment). Dental care also qualifies because it is of interest to both sexes; here, [14] estimate a higher WTP of women for implants, which is confirmed by [15] in the case of extractions, fillings, and cleaning. From surveys, it is known that women have a stronger concern for their health than men [16]. Women’s longer RLE therefore must be combined with their higher WTP in an analysis of the gender difference in HCE.

The second set of predictions (presented and tested in Section 3.2) concerns the age gradient of HCE, which turns out also to depend on the presence (absence, respectively) of rationing. In principle, the age gradient could also be affected by a change of WTP as a function of both age and RLE. Indeed, there is some evidence suggesting that WTP falls with age [17]. However, the authors fail to hold RLE constant, a crucial omission in view of the RHH. In contradistinction, in a recent experiment of the discrete-choice type designed to measure both private and social WTP for an end-of-life medical intervention, age does not prove to be a significant predictor [18]. 

Section 4 offers concluding remarks along with an overview of the outcomes of empirical tests and of the limitations of this study.

## 2. Materials and Methods

### 2.1. Absence and Presence of Rationing in Health Care

The ‘red herring’ hypothesis (RHH) originally was developed without taking a possible effect of rationing into account, which is also true of Figure 1. In the following, the RHH is assumed to be true; rather than trying to derive the age profile of HCE from dynamic optimization as notably in [19]. There, the optimal age profile of HCE can be derived only by a simulation involving parameters that are not reported in the published literature designed to test the RHH (see Table 1 and Table 2). However, the remit of this paper is to pit the RHH against actual empirical evidence.

The starting point is a specification of HCE reflecting the RHH. If HCE is governed by the patient (denoted by $x^{P}$), the hypothesis can be approximated by

(1) $$\begin{array}{l} x^{P}={\left(a-bT\right)}^{2},\;\\ \mathrm{with}\;a>0,\;0<b<a/T\;\mathrm{for}\;\mathrm{all}\;T,\;\partial x^{P}/\partial T=-2b\left(a-bT\right)<\;0,\;\\ \partial^{2}x^{P}/\partial T^{2}=2b^{2}\;>\;0,\;\partial x^{P}/\partial a=2\left(a-bT\right)>\;0,\;\\ \mathrm{and}\;\partial^{2}x^{P}/\partial T\partial a=-2ba\;<\;0,\; \end{array}$$

with T>0: time to death (RLE, respectively). Note that HCE increases with closeness to death (T→0) regardless of age, in keeping with the RHH as defined in Section 1. Due to $\partial^{2}x^{P}/\partial T^{2}>0$, the fall in HCE becomes less marked with increasing distance from death; conversely, HCE increases progressively with proximity of death, as found, e.g., by [1]. Note that this formulation cannot accommodate the observation that sometimes HCE drops in the last year of life. However, with only a couple of exceptions among the studies collected in Table A1 of Appendix B, this is the case when TTD is not controlled for, while a cubic equation would greatly add to the complexity of the analysis in Appendix D and Appendix E. Also, prices and income are neglected since the evidence cited in Section 3.1 and Section 3.2 relates to individuals with a high degree of health insurance coverage. 

As argued in the Introduction, in rationed settings observed HCE is the outcome of demand-side and supply-side influences because physicians do not simply implement the demands of their patients. Yet observed HCE given rationing does not represent the “short side” of the market (in view of comprehensive insurance coverage, price has (almost) no influence) but is the resultant of physicians exerting rationing effort and patients exerting effort to obtain care as desired. A simple structural model is designed to reflect this interaction. On the patient side, the generic utility function.

(2) $$\begin{array}{l} u^{P}=u^{P}\left\{x^{P}\left(e^{P};T\right)\right\}-e^{P}\\ \mathrm{with}\;\;\;\frac{\partial u^{P}}{\partial x^{P}}>\;0,\;\frac{\partial^{2}u^{P}}{\partial x^{P2}}<\;0,\;\frac{\partial x^{P}}{\partial e^{P}}>\;0\;\mathrm{and}\;\frac{\partial^{2}x^{P}}{\partial e^{P2}}<\;0\; \end{array}$$

depends on the amount of medical services $x^{P}$ which in turn increases with patient effort $e^{P}$ (in utility terms) aimed at obtaining the desired amount of care. Of course, this is not to deny that ultimately patients value health. However, for the present purpose, analyzing derived utility as a function of medical services is sufficient. Moreover, the amount of medical services (and utilization of healthcare resources more generally) is not distinguished from HCE for simplicity because countries with their different levels of fees and prices are not directly compared here. The first-order optimality condition reads,

(3) $$\frac{\partial u^{P}}{\partial x^{P}}\frac{\partial x^{P}}{\partial e^{P}}-1=0;\;$$

the interplay of its two components is assumed to give rise to Equation (1). For instance, $\partial u^{P}/\partial x^{P}$ and $\partial x^{P}/\partial e^{P}$ may both decrease with T.

In the case of rationing, the physician (denoted by superscript *R*) is hypothesized to have utility (with $e^{R}$ symbolizing rationing effort and A, patient age),

(4) $$\begin{array}{l} u^{R}=u^{R}\left\{x^{R}\left(e^{R};T,A\right)\right\}-e^{R}\;\mathrm{if}\;\frac{\partial u^{R}}{\partial x^{R}}>0,\\ \mathrm{with}\;\frac{\partial^{2}u^{R}}{\partial x^{R2}}<0,\;\frac{\partial x^{R}}{\partial e^{R}}<0,\;\frac{\partial^{2}x^{R}}{\partial e^{R2}}>0;\; \end{array}$$

(5) $$\begin{array}{l} u^{R}=u^{R}\left\{x^{R}\left(e^{R};T,A\right)\right\}+e^{R}\;\mathrm{if}\;\frac{\partial u^{R}}{\partial x^{R}}<0,\\ \mathrm{with}\;\frac{\partial^{2}u^{R}}{\partial x^{R2}}<0,\;\frac{\partial x^{R}}{\partial e^{R}}<0,\;\frac{\partial^{2}x^{R}}{\partial e^{R2}}>0. \end{array}$$

In a rationed setting, physicians may still prefer to provide more care to patients to avoid a bad conscience [20]. In that event $\partial u^{R}/\partial x^{R}>0$, with $x^{R}$ symbolizing the resulting HCE, and their rationing effort amounts to a deduction of their utility as in Equation (4). However, some of them may derive utility from satisfying the demands of authorities if they are compensated for their effort, again in utility terms. In this event Equation (5) applies with $\partial u^{R}/\partial x^{R}<0$ and $\partial^{2}u^{R}/\partial x^{R2}<0$. In both cases, more rationing effort gives rise to less HCE, with decreasing marginal effectiveness. 

The optimality conditions for an interior solution read,

(6) $$\frac{\partial u^{R}}{\partial x^{R}}\frac{\partial x^{R}}{\partial e^{R}}-1=0;\;$$

(7) $$\frac{\partial u^{R}}{\partial x^{R}}\frac{\partial x^{R}}{\partial e^{R}}+1=0.$$

In Appendix A, the outcome of the interaction between the patient and the rationing physician is modeled as a Nash equilibrium in terms of efforts $e^{P}$ and $e^{R}$ which also determines HCE in equilibrium. However, these effort levels are unobservable. On the patient’s side, effort is driven by maximum willingness to pay a, the parameter b, and time to death T if the RHH is true (see Equation (1) again). On the physicians’ side, the determinants of their utility continue to be an open issue according to the reviews by [21,22]. As to rationing in particular, [23] note in their international review that most of it is implicit rather than explicit, which also means that the criteria applied vary greatly. However, two are mentioned with high frequency, (cost) effectiveness and equity. The first is satisfied to a greater degree *ceteris paribus* if patients have more years to live beyond their current age. Equity as well as the ‘fair inning’ argument of [24] call for linking the amount of care the rationing physician is willing to provide to remaining life expectancy adjusted for age. A tractable specification is T/A, which reflects age-based rationing, as proposed by [25] and extensively discussed in [26]. Finally, [27] find evidence suggesting that physicians treat patients with longer remaining life expectancy more aggressively than others. Therefore, assume that the unobservable optimality conditions (6) and (7) give rise to the following function in observable quantities,

(8) $$x^{R}=f+g\cdot \left(T/A\right),\;\mathrm{with}\;f>0,\;g>0.$$

**Figure A1:**
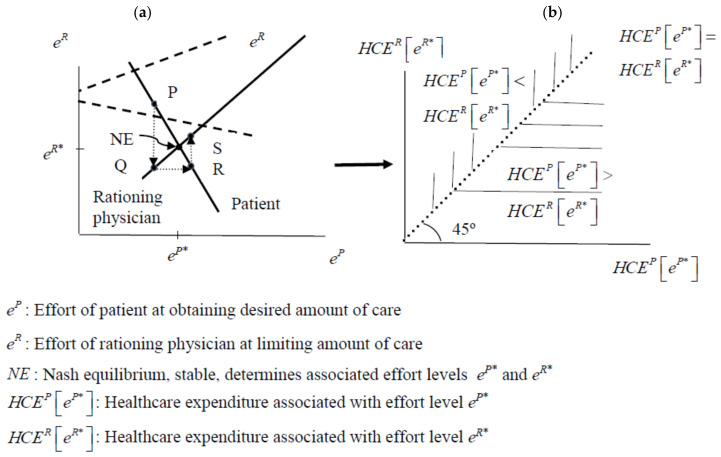
Outcomes associated with the patient-physician interaction given rationing. (**a**) in efforts space (dashed no equilibrium; (**b**) in HCE space.

At a Nash equilibrium with positive HCE (see Figure A1 of Appendix A), one has in view of Equations (1) and (8),

(9) $$x\left[e^{P}*\right]=x\left[e^{R}*\right],\;\mathrm{implying}\;{\left(a-bT\right)}^{2}=f+g\cdot \left(T/A\right),\;$$

with $e^{P}*$ and $e^{R}*$ symbolizing optimal effort levels in response to that of the other player. 

This simple formulation could be criticized on the grounds that physicians act as Stackelberg leaders rather than being on a par with their patients. However, a Stackelberg solution would require explicit modeling of physician behavior (to derive iso-utility curves). In view of the uncertainty surrounding medical objectives cited above, this approach would rest on shaky theoretical foundations. Therefore, the symmetric Nash equilibrium derived in Appendix A is retained. 

### 2.2. Categorization of the Evidence to Be Discussed According to the Presence and Absence of Rationing 

This section is devoted to the sources of empirical evidence regarding the ‘red herring’ hypothesis to be discussed in Section 3.1 and Section 3.2. While they originally comprised almost 30 publications, those assembled in Table A1 of Appendix B had to satisfy the following requirements:

- The publication relates to the ‘red herring’ hypothesis in one way or another yet need not explicitly be designed to test it;
- The evidence presented is sufficiently detailed to permit a test of at least two predictions derived either from Appendix D (gender difference) or Appendix E (age profile of HCE);
- The author of this paper is/was not involved in the research.

Countries and settings are categorized according to whether or not their healthcare sector is subject to rationing. Admittedly, this categorization reflects the subjective judgment of the authors cited for testimony in Table A1 at least to some extent. Out of the 15 publications retained, 12 (=80 percent) support the RHH according to the authors’ judgment, with six (Nos. 2, 6, 8, 9, 12, and 14) coming from a country or setting with at least some rationing. Conversely, two of the three studies that do not support the RHH relate to countries (or sets of countries) with at least some rationing. Therefore, while the RHH initiated from a country without rationing (Switzerland), it may apply more generally provided physicians’ rationing effort is accounted for.

**Conclusion** **1.** Out of the many studies revolving around the ‘red herring’ hypothesis published since its launch in 1999, 15 permit testing the hypothesis in at least two ways with regard to women’s longer remaining life expectancy or the age profile of healthcare expenditure. A clear majority comprising countries and settings both without and with rationing finds supporting evidence.

## 3. Results

### 3.1. The Effect of Women’s Longer Remaining Life Expectancy on HCE with and without Rationing (W-Predictions)

#### 3.1.1. Statement of W-Predictions

Throughout this section, women are characterized by their higher RLE and their higher WTP for healthcare services compared to men (denoted by dT>0 and da>0, respectively in Equation (1); a possible difference in *b* is neglected for simplicity). Note that although the gender differences in *T* and *a* are more than marginal, *dT* and *da* rather than Δ*T* and Δ*a* are used to keep notation simple. In Appendix D, the predictions concerning the induced gender difference in HCE are derived from the models of Section 2.1; they appear in Table 1 below. 

As to WNR1 (no rationing, Equation (A7)), women’s higher WTP dominates the effect of their greater distance from death *T*, resulting in higher HCE in the general population. The reason is that patients’ WTP is found to vary little with distance from death in Appendix C (the parameter *b* in Equation (1) is clearly below one). 

Prediction WR1 (rationing, Equation (A11)) reflects the interaction between the patient and the rationing physician. Accordingly, all the parameters appearing in Equation (9) play a role. In particular, with b<1 in the general population, women’s higher WTP (da>0) again dominates their greater distance from death, causing their HCE to exceed that of men.

Turning to WNR2 (no rationing, Equation (A7)), one has to take into account that in the year prior to death at the latest, b<1, causing their higher RLE to lose influence and leading to the prediction that their HCE is higher than men’s, with the difference depending positively on current HCE which reflects their WTP. The same prediction holds according to WR2 (rationing, Equation (A11)): Here, the difference depends negatively on patient age due to the physician’s rationing influence.

According to WNR3 (no rationing, Equation (A8)), a decreasing RLE (increasing closeness to death, respectively) has a weaker increasing effect on women’s HCE than men’s in the general population. A change in RLE (with the difference in favor of women held constant) affects the two genders in the same way by assumption. Since b<1 (see Appendix C), its impact is small, hence a comparatively weak influence of the proximity of death on women’s HCE resulting in a lower value compared to men. This is also the prediction of WR3 (rationing, Equation (A12)), although the reason is different. Here, a change in RLE greatly affects the amount of care the physician is willing to provide, especially to women with their higher RLE (see $T^{2}$ in Equation (A12) of Appendix D). This means that their HCE increases more slowly than men’s with proximity to death; yet the difference is small because the physician takes into account patient age (which is usually high). 

As to WNR4 (no rationing, Equation (A8)), the parameter b exceeds one until shortly before death (see Appendix C), resulting in a stronger effect on women’s HCE compared to men’s with an increase in their RLE. Therefore, their HCE increases at a lower (constant) rate with closeness to death (recall the negative value of Equation (A7)). The prediction WR4 (rationing, Equation (A13)), is the same, but again for a different reason: Now the parameters in T appearing in Equation (A12) go to zero, causing its originally strong influence on the gender difference in HCE to be reduced.

Finally, WNR5 (no rationing, Equation (A9)) relates the development of HCE over time, which is assumed to be driven by an increase in maximum WTP due to new medical technology and resulting in an increase in RLE for both sexes (da>0 and dT>0, with the initial differences in favor of women again held constant). Since these changes are assumed to affect both genders in the same way while physicians do not intervene, the predicted HCE difference in favor of women is unaffected regardless of closeness to death. According to WR5 (rationing, Equation (A14)), however, the physician is willing to consent to more HCE in response to higher RLE and hence effectiveness of medical care regardless of gender, which leads to a convergence of women’s and men’s HCE over time (especially at very high age, again because the physician takes patient age into account).

A comparison between the two columns of Table 1 below reveals that the presence (absence, respectively) of rationing does make a clear difference in two out of five instances. Whereas WNR2 (no rationing) predicts that women close to death exhibit higher HCE than men in the same situation, WR2 (rationing) predicts it to be lower than men’s. Regarding the development of HCE over time, WNR5 predicts a constant difference in favor of women, while according to WR5, any difference tends to vanish, with full convergence at very high age. In the remaining three cases, predictions differ in detail only, making testing difficult.

**Table 1:** Predictions and evidence regarding women’s HCE compared to men’s under the ‘red herring’ hypothesis (RHH).

No Rationing		Rationing	
Prediction; Source ^1^	Confirmed? ^2^	Prediction; Source ^1^	Confirmed ? ^2^
WNR1: In the general population, women exhibit higher HCE than men, with the difference depending positively on current HCE; Equation (A7)	Hashimoto et al. (2020) [28]: ? Karlsson et al. (2016) [29]: y Moorin et al. (2012) [30]: y	WR1: In the general population, women exhibit higher HCE than men, with the difference depending negatively on patient age; Equation (A11)	Costa-Font and Vilaplan-Rieto (2020) [31]: y Gregersen (2013) [32]: p Howdon and Rice (2018) [33]: ? Lorenz et al. (2020) [34]: y Seshamani and Gray (2004) [35]: ? Wei and Zhou (2019) [36]: n
WNR2: In their last year before death at the latest, women exhibit higher HCE than men, with the difference depending positively on current HCE; Equation (A7)	Hashimoto et al. (2020) [28]: y Karlsson et al. (2016) [29]: y Moorin et al. (2012) [30]: n	WR2: In their last year before death at the latest, women exhibit lower HCE than men, with the difference depending negatively on patient age; Equation (A11)	Costa-Font and Vilaplan-Rieto (2020) [31]: n Gregersen (2013) [32]: y Howdon and Rice (2018) [33]: y Lorenz et al. (2020) [34]: y Seshamani and Gray (2004) [35]: n Wei and Zhou (2019) [36]: ?
WNR3: In the general population, women’s HCE increases at a lower constant rate than men’s with closeness to death; Equation (A8)	Hashimoto et al. (2020) [28]: ? Karlsson et al. (2016) [29]: ? Moorin et al. (2012) [30]: y	WR3: In the general population, women’s HCE increases at a lower rate than men’s with closeness to death, with the difference depending negatively on patient age; Equation (A12)	Costa-Font and Vilaplan-Rieto (2020) [31]: n Gregersen (2013) [32]: ? Howdon and Rice (2018) [33]: ? Lorenz et al. (2020) [34]: n Seshamani and Gray (2004) [35]: ? Wei and Zhou (2019) [36]: ?
WNR4: In their last year before death at the latest, women’s HCE increases at a lower constant rate than men’s with closeness to death; Equation (A8)	Hashimoto et al. (2020) [28]: p Karlsson et al. (2016) [29]: p Moorin et al. (2012) [30]: y	WR4: In their last year before death at the latest, women’s HCE increases at a rate slightly lower than men’s with closeness to death, with the difference depending negatively on patient age; Equation (A13)	Costa-Font and Vilaplan-Rieto (2020) [31]: y Gregersen (2013) [32]: ? Howdon and Rice (2018) [33]: y Lorenz et al. (2020) [34]: y Seshamani and Gray (2004) [35]: y Wei and Zhou (2019) [36]: ?
WNR5: Any difference between women’s and men’s HCE remains constant over time; Equation (A9)	Hashimoto et al. (2020) [28]: ? Karlsson et al. (2016) [29]: ? Moorin et al. (2012) [30]: y	WR5: Women’s HCE approaches that of men over time, converging at very high age; Equation (A14)	Costa-Font and Vilaplan-Rieto (2020) [31]: ? Gregersen (2013) [32]: y Howdon and Rice (2018) [33]: ? Lorenz et al. (2020) [34]: y Seshamani and Gray (2004) [35]: y Wei and Zhou (2019) [36]: ?
**Totals**	**y: 7; p: 2; n: 2; ?: 5**		**y: 12; p: 1; n: 4; ?: 11**

^1^ The equation number refers to the pertinent Appendix; e.g., (A16) to Appendix D. ^2^ y: yes; p: partial; n: no; ?: no test possible.

#### 3.1.2. The W-Evidence

The papers to be discussed are listed in Table A1 of Appendix B. They are evaluated with respect to the effect of women’s longer remaining life expectancy RLE (time to death TTD, respectively) on their HCE compared to men’s.

*Costa-Font and Vilaplan-Rieto* (2020) [31], mostly rationing

The authors use the Survey for Health, Ageing and Retirement in Europe (SHARE) dataset covering the years 2004 to 2017 (waves no. 1 to 7, with no. 3 excepted) and 17 countries. According to their Table 1, the majority of individuals sampled (and observations) are from countries that impose rationing (even though the United Kingdom is not included). Their sample includes some 54,500 individuals aged 50+ of which 2760 died. In order to control for the endogeneity of TTD with respect to healthcare services, the authors use parents’ ages of death as instruments, estimating the values of living parents through multiple imputation. Rather than HCE, they analyze (a) the likelihood of and (b) the length of stays in hospital, outpatient visits, stays in nursing home, personal care, and prescription drugs consumed. They find support of the RHH throughout. However, somewhat contrary to the paper’s concluding sentence, “The effect of ageing on health care use seems to be simultaneously affected by several red herrings”, the estimates with endogenous TTD presented in the author’s Table 3 reveal that components (b) have similar coefficients pertaining to *Age*, *Age^2^*, and TTD. Since outpatient visits are the component of HCE where patient demand most likely interacts with rationing effort on the part of the physician, the discussion focuses on this variable. 

In their Figure E.2 (panel d), the authors display the age gradients of outpatient visits with TTD > 36 as well as TTD = 12 months. If one is willing to count the age group 50–64 as being still part of the general population, WR1 is confirmed as women exhibit more visits than men. However, WR2 is contradicted because the difference in favor of women obtains also when TTD = 12 months. Additionally, the rate of change is not higher among women than men, contradicting WR3. Yet women’s visits do display a (weak) tendency towards convergence with men’s when TTD decreases, vindicating WR4. Finally, WR5 cannot be tested because the SHARE waves are aggregated. 

*Gregersen* (2014) [32], Norway, some rationing 

This study uses hospital data, covering all admissions 1998–2009 recorded by the (very comprehensive) Norwegian Patient Registry. While the author’s regression analysis reveals a spike in HCE during the last year of patients’ lives, this spike decreases with age. Still, the author concludes that his findings support the RHH (see Table A1).

As to prediction WR1, the female/male HCE ratio can be read off from the author’s Figure 1. At age 40 (beyond normal childbearing age), it is 1.33 in 1998–2003 and increases up to age 50. By age 70, however, it is 0.52 (a similar reversal holds in the period 2004–2009). This constitutes but partial confirmation. Beyond age 80, females consistently exhibit lower HCE than males, confirming prediction WR2. 

Predictions WR3 and WR4 cannot be tested because the author does not vary TTD.

As to prediction WR5, women’s life expectancy in Norway increased from 81 years in 1998 to 83 years in 2009 [37], arguably also reflecting access to new medical technology. In the authors’ Figure 1, the female/male HCE ratio increases from 1.50 at age 40 (after childbearing age) in 1998–2003 to 1.57 in 2004–2009; at age 70, it increases from 0.75 and 0.83. Finally, this ratio was 1.00 in 2004–2009, indicating convergence between gender-specific HCE and hence full vindication of RW5. 

*Hashimoto* et al. (2010) [28], Japan, no rationing

The authors use data on beneficiaries of National Health Insurance aged 65+ in the southern Kyushui district, some 51,000 of whom died between 2001 and 2003 while 365,000 were alive in 2004 so had TTD ≥ 12 months. Recorded HCE comprises outlays on outpatient, inpatient, home care, and institutional care. The authors find support of the RHH. 

The authors’ Table 2 exhibits the four components of HCE for males and females in the age groups 65–74, 75–84, and 85+, (too high for testing WNR1) distinguishing between survivors and decedents. Prediction WNR2 applies to this latter category; it is confirmed, being supported in 10 out of 12 comparisons. Only in the age group 75–84 is women’s homecare expense higher than men’s and in the age group 85+, for institutional care. Prediction WNR4 is partially confirmed, being vindicated in eight out of 12 comparisons distinguishing between survivors and decedents (who evidently were closer to death). With increasing closeness to death, women indeed exhibit a more moderate increase in HCE than men; the four exceptions again relate to homecare and institutional care. 

Finally, WNR5 cannot be tested because the three years of observation are lumped together. 

*Howdon and Rice* (2018) [33], England, rationing 

The authors’ data set covers the financial years 2005/06 to 2011/12; it is split in two samples comprising some 40,000 individuals aged 50+ each. In the first sample, individuals died 2011/12; in the second, they died between 2005/06 and 2010/11. The authors conclude that the RHH is confirmed in that TTD rather than age drives HCE but emphasize that TDD itself is a proxy for morbidity on which they have detailed information. Indeed, in their regressions the coefficient pertaining to *log*(*TTD*) typically drops by two-thirds when morbidities are included.

With observations starting at age 50, predictions WR1 and WR3 cannot be tested. As to WR2, a comparison of the authors’ Figures 3 and 4 reveals that men’s HCE in the last four quarters before death indeed exceeds that of women (although confidence intervals are not given), confirming the prediction. Additionally, the increase in women’s HCE between quarters no. 16 and 4 before death is indeed slower than men’s, as predicted by WR4. 

Prediction WR5 cannot be tested since the authors do not report the comparisons performed above for their two samples.

*Karlsson* et al. (2016) [29], German private health insurer, no rationing

In this work, some 600,000 persons were observed from 2005 to 2011; being privately insured, they are not subject to rationing (Sections 3.4 and 3.5 of the authors’ text). They find that annual HCE increases strongly between ages 50 and 80 and that with 5.6 percent, a relatively low share of lifetime HCE occurs in the last year of life. Yet, noting that the last three years of life account for almost 14 percent of lifetime HCE, they do not deem the RHH to be rejected (see Table A1). The authors also note a high degree of persistence over time in that the probability of being in the same quintile of the HCE distribution is at least 0.5 over six years.

Prediction WNR1 is confirmed because females consistently exhibit higher HCE than men; moreover, the difference increases with higher HCE, with two exceptions in the 65+ age bracket (authors’ Table 5). Prediction WNR2 receives full support. According to the authors’ Table 9, the HCE ratio in favor of women is 1.34 two years before death and 1.37 in the last year before death. Additionally, this increase in the ratio goes along with an increase in current HCE, as predicted. 

WNR3 cannot be tested because the authors do not exhibit the development of HCE during the last six years of life according to gender in their Figure 11. As to WNR4, women’s HCE increases by a mere 0.5 percent from two years to one year before death, compared to 3 percent of men’s. However, the constancy of these increases cannot be verified with just two years, resulting in partial support. WNR5 cannot be tested either since the authors do not report changes in HCE between 2005 and 2011. 

*Lorenz* et al. (2020) [34], Germany, rationing

The authors on average have observations on about 320,000 individuals covered by social health insurance over the period from 2001 to 2015, during which some 34,400 women and 30,000 men died. Estimating third-order polynomials, they derive age-specific trends in real expenditure, distinguishing between “ordinary” HCE and long-term care expenditure. Since age continues to have a positive impact on HCE even when TTD is controlled for, they conclude that their evidence contradicts the RHH.

However, WR1 is confirmed since in the authors’ Figure 2 women’s HCE exceeds men’s between ages 15 and 50, i.e., well beyond childbearing age, with the difference increasing at first but then decreasing with age, as predicted. As to WR2, it is confirmed as well because regardless of TTD (which is varied between one and four years in the authors’ Figure 3), women exhibit higher HCE than men across all ages. 

However, WR3 is contradicted because from four years to one year before death, women’s HCE increases by a factor of about 4.5 at age 40 (at earlier ages, confidence intervals in the authors’ Figure 3 are very wide, causing values to overlap), men’s, by a factor of about 3.4 only. At age 50, women’s HCE multiplier is as high as 5.0, and men’s about 3.75 only. Therefore, women’s HCE increases faster than men’s—and the difference widens rather than narrows with age. As to prediction WR4, it is confirmed. The HCE multiplier from just two years to one year before death is only 1.54 at age 50 for women but 2.61 for men. At age 60, the two multipliers drop to 2.47 and 2.67, respectively, at age 70, to 2.29 and 2.48; at age 80, they are 2.0 and 2.48, respectively. Therefore, women’s HCE does increase at a lower rate than men’s with closeness to death, with the difference between the two rates largely decreasing with age, as predicted.

Prediction WR5 is confirmed by the authors’ Figure 5a, which displays growth rates of real HCE in the last year of life. Whereas up to age 76 women’s HCE increases at rates sometimes faster and sometimes slower than men’s, by the age of 85 its growth rate cannot be distinguished from that of men, indicating the predicted convergence. 

*Moorin* et al. (2012) [30], Western Australia, no rationing

In their attempt to test the RHH, the authors limit their database to the 60,498 individuals who died between 1990 and 2004. They subdivide this period of observation in three eras: 1990–1994 with 22,143 decedents (era 1), 1995–1999 with 19,756 (era 2), and 2000–2004 with 18,599 decedents (era 3). In addition, they distinguish HCE on (a) primary care, (b) specialist, and (c) diagnostic and therapeutic services. The authors are in broad support of the RHH (see Table A1).

As to prediction WNR1, in category (a), women’s HCE exceeds that of men across all ages in all three eras and in categories (b) and (c), in all three eras up to age 70. In the three out of nine cases [three eras, categories (a), (b), and (c)] where HCE increases with age, the difference in HCE increases as well; in the six cases where HCE decreases with age beyond childbearing age, the difference in HCE decreases, too, establishing the predicted positive correlation and providing clear support of WNR1. As to WNR2, it is contradicted in that in seven out of nine cases displayed in the authors’ Figure 1, women at high age exhibit lower (fitted) HCE than men. 

Prediction WNR3 is confirmed in all nine instances; in era 1, e.g., the HCE ratio in women’s favor was approximately 1.90 at age 40 but dropped to 1.42 at age 80, reflecting a slower increase than among men with closeness to death at a roughly constant rate (see the authors’ Figure 1 after enlargement). 

As to WNR4, in their Figure 2 the authors plot HCE at 60 to 37, 36 to 25, 24 to 13, 12 to 3, and 2 to 0 months before death. In eight of nine cases, women’s HCE increase at a lower rate than men’s, as predicted. With the exception of category (b), HCE increases progressively with closeness to death in all three eras, as predicted by the RHH. 

As to prediction WNR5, women in Australia arguably had access to improved medical technology as in other industrial countries, and they saw their RLE increase, too. In 1960–1962, RLE was 16 years at age 65 and increased to 22 years by 2011–2013 [38]. Across the three eras distinguished, the difference between women’s and men’s HCE displays an almost perfect constancy, as predicted. 

*Seshamani and Gray* (2004) [39], United Kingdom, rationing

Designed explicitly to test the RHH, this study uses hospital data covering some 91,000 admissions of patients aged 65 and higher from 1977 to 1999. It finds that HCE starts rising as early as 15 years prior to death but increases tenfold during the last five years of life. This boost exceeds the 30 percent increase between ages 65 and 85, leading the authors to conclude that the RHH is vindicated (see Table A1).

The authors report HCE only after age 65, obviating a test of WR1. Their Table 4 shows consistently higher HCE for females than males, contradicting WR2. 

Prediction WR3 cannot be tested either. As to WR4, the authors’ Table 4 exhibits HCE ten years and one year prior to death, respectively, making a proximate test possible. Among women, this difference is associated with an extra HCE of GBP 2967 at age 65 and GBP 3061 at age 95, a minimal increase. Among men, the HCE difference is GBP 2292 at age 65 but rises to GBP 2657 at age 95. Therefore, closeness to death is associated with a lower increase in women’s HCE than men’s, with the difference decreasing past the age of 75, thus confirming R4. 

Prediction WR5 can be tested as well. Women’s life expectancy increased from 75 years in 1970–1972 to 79 years in 1990–1992 [40], suggesting that the National Health Service of the United Kingdom granted citizens access to new medical technology as in other countries. The authors’ Figure 7 distinguishes the years 1970, 1980, and 1990 and ages 90, 95, and 100. The female/male HCE ratio falls from 1.21 in 1970 to 0.94 in 1990 for women aged 90, indicating convergence. At the very high age of 95, it falls from 1.39 in 1970 to 0.91 in 1990, confirming WR5 once again.

*Wei and Zhou* (2020) [36], China, rationing 

Whereas in private correspondence the first author claims there is no rationing in China, arguing that the (mandatory) benefit list of health insurance is comprehensive, the paper explains the drop in hospitalizations and HCE after age 60 among the deathbound by a “more conservative treatment … for the elderly”, likely reflecting age-based rationing.

The authors use the 2011 and 2013 waves of the China Health and Retirement Longitudinal Study (CHARLS) to determine TTD for the 401 individuals (out of some 17,500 aged 45+) who died between the two years. They find support for the RHH since *Age* as well as *Age*^2^ lose statistical significance as soon as TTD1 to TTD3 (=1 if deceased in the first (second, last year after 2011)) are included in the regression for *lnHCE*. While they do not systematically distinguish between men and women, the male dummy in their estimates for the whole sample and the subsample of 60+ olds permits to test two of the W-hypotheses. 

In fact, in the author’s Table 2 (which refers to individuals aged 45+, roughly still the general population), the male dummy points to HCE that is approximately 30 percent lower for men than for women, clearly confirming WR1. As to WR2, their Table 3 refers to individuals aged 60+ (analyzed in their Table 2) who can be said to be somewhat close to death on average, Chinese life expectancy being 75 years in 2011 (https://data.worldbank.org/indicator/SP.DYN.LE00. IN?locations = CN, accessed on 15 November 2021). However, estimated HCE once again points to a 30 percent lower value among men, contradicting WR2.

Predictions WR3 throughWR5 cannot be tested either because the evidence is not presented separately for women and men.

These findings (see Table 1 again) give rise to

**Conclusion** **2.** In the 28 instances where the published results are sufficiently detailed to permit testing with regard to the gender difference in HCE, in the case of no rationing seven out of eleven are in full and one in partial support of the ‘red herring’ hypothesis. In the case of rationing 12 out of 17 are in full and one in partial support. As to the two cases where the presence (absence, respectively) of rationing makes a difference, both WR2 and WNR2 are confirmed five out of nine times.

### 3.2. The Age Profile of HCE under the ‘Red Herring’ Hypothesis with and without Rationing (A-Predictions)

#### 3.2.1. Statement of A-Predictions

For simplicity, the difference in HCE induced by women’s longer RLE is neglected in this section the better to focus on the effects of age and aging. However, in view of the RHH an important distinction is whether or not these effects are stated with remaining life expectancy RLE (time to death TTD, respectively) held constant. Whereas ANR1 (no rationing, Equation (A15) of Appendix E)) predicts an increase in HCE with age because death draws closer, it predicts constancy if TTD is controlled for, in accordance with the RHH. Yet AR1a (rationing, Equation (A19)) predicts a decrease in HCE with age when TTD is not held constant as long as patients are of young to medium age. Physician influence causes HCE to decrease with patient age *ceteris paribus*, an effect which dominates at young to medium age because patients’ WTP is low when death is still far away. 

According to AR1b (Equation (A19)), the relationship between patient age and HCE turns positive at high age since the physician’s rationing effort is governed by the ratio of RLE to age, a ratio which now is close to zero so loses its impact. The interaction between the two players then boils down to one between the patient’s increasing WTP with closeness to death and the physician’s basic willingness to provide medical care; this results in HCE increasing with age. 

Turning to the case where RLE is held constant, one has ANR2 (no rationing, Equation (A16)) simply stating that HCE does not vary with age since patients respond to TTD only. By way of contrast, AR2a (rationing, Equation (A21)) predicts that HCE falls with patient age in the general population, at a rate which decreases with age due to age-based rationing whose effect becomes ever more dominant. However, according to AR2b (rationing, Equation (A22), the age profile of HCE becomes flat at very high age, once again because the ratio of RLE to patient age approaches zero so loses its importance to the physician. 

Finally, a steepening of the age profile in HCE over time is predicted by ANR3 (no rationing, Equation (A17)) in the general population. There, patients’ WTP does not decrease strongly with the increase in RLE yet; therefore, the increase in WTP in response to new medical technology dominates. Steepening over time is also predicted by AR3 (rationing, Equation (A22)), albeit at a rate that depends negatively on patient age due to the physician’s influence. In the last year before death at the latest, when patients’ WTP falls markedly with an increase in TTD, ANR4 (no rationing, Equation (A17)) predicts a flattening of the age profile of HCE in response to this increase. This holds also for AR4 (Equation (A22), and for the same reason (b>1) because the influence of the physician becomes relatively stronger, although with lowered rationing effort due to increased TTD. 

Once again, the presence (absence, respectively) of rationing matters. First of all, in the absence of rationing there are but four predictions (ANR1 to ANR4), while a rationing context gives rise to six (AR1a, AR1b, AR2a, AR2b, AR3, AR4) due to the importance of age in rationing. Next, ANR1 predicts an increase in HCE with patient age if TTD is not held constant but AR1a predicts a decrease at young to medium and patient age (which turns into an increase at high age according to AR1b). The remaining predictions (ANR3 vs. AR3, ANR4 vs. AR4) differ in detail only.

#### 3.2.2. The A-Evidence 

*Bjørner and Arnberg* (2012) [41], Denmark, some rationing

This study is based on some 500,000 individuals per year who were observed between 2000 and 2009. According to the authors, it is in support of the RHH.

As to prediction AR1a, the components of HCE (hospital, psychiatry, medicine, GPs, and specialists) all decrease with age between ages 0 and 14 as well as 30 and 42 when TTD is not held constant up to age 85 and at a high rate between ages 15 and 32 (authors’ Figure 1), but at rates that do not consistently increase with current HCE. Therefore, support of AR1a is but partial. AR1b is also confirmed in part only because the components HCE increase progressively between ages 60 and 80—but decrease beyond age 86. 

In the authors’ Figure 2, total HCE is related to values of TTD ranging in five steps from one to nine and more years (the latter roughly reflecting the general population cited in AR2a). However, the predicted decrease is observed between ages 30–34 and 40–44 only, providing but partial support of AR2a. Prediction AR2b is also partially confirmed in that beyond the age group 80–84 three out of the five age profiles become flat, while two are even decreasing.

**Table 2:** Predictions and evidence regarding the age profile of HCE under the ‘red herring’ hypothesis (RHH) ^1^.

No Rationing		Rationing	
Prediction; Source ^1^	Confirmed? ^2^	Prediction; Source ^1^	Confirmed? ^2^
ANR1: If RLE is *not* held constant, HCE increases with patient age at a rate that depends positively on current HCS; Equation (A15)	De Nardi et al. (2016) [42]: y Hashimoto et al. (2010) [28]: y Karlsson et al. (2016) [29]: y Karlsson et al. (2020) [43]: y Moorin et al. (2012) [30]: ?	AR1a: If RLE is *not* held constant and at young to medium age, HCE decreases with patient age; Equation (A19)	Bjørner and Arnberg (2012) [41]: p Costa-Font and Vilaplan-Rieto (2020) [31]: ? Geue et al. (2014) [44]: y Gregersen (2014) [32]: n Hazra et al. (2017) [45]: ? Kolodziejczyk (2020) [46]: ? Lorenz et al. (2020) [34]: p
--	--	AR1b: If RLE is *not* held constant and at high age, HCE increases with patient age; Equation (A19)	Bjørner and Arnberg (2012) [41]: p Costa-Font and Vilaplan-Rieto (2020) [31]: y Geue et al. (2014) [44]: n Gregersen (2014) [32]: y Hazra et al. (2017) [45]: y Kolodziejczyk (2020) [46]: ? Lorenz et al. (2020) [34]: y
ANR2: If RLE *is* held constant, HCE does not vary with age; Equation (A16)	De Nardi et al. (2016) [42]: ? Hashimoto et al. (2010) [28]: p Karlsson et al. (2016) [29]: ? Karlsson et al. (2020): [43]: n Moorin et al. (2012) [30]: p	AR2a: If RLE *is* held constant, HCE in the general population falls with patient age at a rate that depends negatively on patient age; Equation (A20)	Bjørner and Arnberg (2012) [41]: p Costa-Font and Vilaplan-Rieto (2020) [31]: ? Geue et al. (2014) [44]: ? Gregersen (2014) [32]: n Hazra et al. (2017) [45]: ? Kolodziejczyk (2020) [46]: n Lorenz et al. (2020) [34]: y
--	--	AR2b: If RLE *is* held constant and at very high age, the age profile of HCE becomes flat; Equation (A21)	Bjørner and Arnberg (2012) [41]: p Costa-Font and Vilaplan-Rieto (2020) [31]: y Geue et al. (2014) [44]: y Gregersen (2014) [32]: p Hazra et al. (2017) [45]: y Kolodziejczyk (2020) [46]: y Lorenz et al. (2020) [34]: y
ANR3: In the general population, the age profile of HCE becomes steeper over time; Equation (A17)	De Nardi et al. (2016) [42]: y Hashimoto et al. (2010) [28]: ? Karlsson et al. (2016) [29]: y Karlsson et al. (2020) [43]: ? Moorin et al. (2012) [30]: p	AR3: In the general population, the age profile of HCE becomes steeper over time, with the rate of increase depending negatively on patient age; Equation (A22)	Bjørner and Arnberg (2012 [41]): p Costa-Font and Vilaplan-Rieto (2020) [31]: ? Geue et al. (2014) [44]: ? Gregersen (2014) [32]: p Hazra et al. (2017) [45]: ? Kolodziejczyk (2020) [46]: ? Lorenz et al. (2020) [34]: p
ANR4: In the last year before death at the latest, the age profile of HCE becomes flatter over time; Equation (A17)	De Nardi et al. (2016) [42]: ? Hashimoto et al. (2010 [28]: ? Karlsson et al. (2016) [29]: ? Karlsson et al. (2020) [43]: ? Moorin et al. (2012) [30]: p	AR4: In the last year before death at the latest, the age profile of HCE becomes flatter over time, with the rate of change depending negatively on patient age; Equation (A22)	Bjørner and Arnberg (2012) [41]: ? Costa-Font and Vilaplan-Rieto (2020) [31]: ? Geue et al. (2014) [44]: ? Gregersen (2014) [32]: ? Hazra et al. (2017) [45]: ? Kolodziejczyk (2020) [46]: ? Lorenz et al. (2020) [34]: y
**Totals**	**y: 7; p: 4; n: 1; ?: 7**		**y: 11; p: 9; n: 3; ?: 13**

^1^ The equation number refers to the pertinent Appendix; e.g., (A22) to Appendix E. ^2^ y: yes; p: partial; n: no; ?: no test possible.

As to prediction AR3, the evidence is somewhat indirect. In their Figure 6, the authors compare the predicted contribution of aging to the long-term development of HCE in different scenarios. Given healthy aging (which arguably reflects medical innovation in the course of time), HCE rises at an increasing rate, reflecting a steepening of the age profile of HCE since the authors hold all other influences constant. However, the rate of change cannot be related to patient age; thus, support of AR3 is but partial. 

Prediction AR4 cannot be tested because it relates to the last year before death at the latest, a period not singled out by the authors.

*Costa-Font and Vilaplan-Rieto* (2020) [31], mostly rationing

The database of this paper is described in Section 3.1.2, where the selection of the number of outpatient visits for testing is justified. 

Since observations start at age 50, the ratio of Age to TTD (T/A in Equation (A19) of Appendix E) is below one; therefore, predictions AR1b and AR2b apply. As to AR1b, it is confirmed by the estimate M1 in the authors’ Table 3 which exhibits a positive coefficient pertaining to *Age* while that of *Age*^2^ is insignificant. In the estimate M5 with TTD held constant, *Age* and *Age*^2^ lose their significance (contrary to TTD), implying the flat age profile predicted by AR2b. 

A possible steepening of the age gradient of HCE over time (AR3, AR4) cannot be tested because the waves of SHARE are aggregated.

*De Nardi* et al. (2016) [42], US Medicare, no rationing

The authors measure HCE of 67,000 US Medicare enrollees over the years 1996 to 2010, all at least 65 years old. They find support of the RHH (see Table A1 of Appendix B) since HCE increases markedly during the last 12 months of life. 

The authors’ Figure 3 supports prediction ANR1 because HCE increases with age over an age span of 35 years, with the exception of just four years. Predominantly, the rate of growth goes up with age (and hence HCE), as predicted. However, ANR2 cannot be tested because the authors do not report the age profile of HCE with TTD held constant. 

Prediction ANR3 is confirmed. The authors report the change over three years in the cumulative distribution functions (cdfs) defined over total HCE, HCE excluding nursing homes, and hospitals (in a US context, medical technology is likely to have advanced even over this short time period). In the authors’ Figure 2, the cdfs shift upward, with the amount of shift depending positively on HCE (except for extremely high values of HCE). Because such high values are typical for patient shortly before death, this absence of a shift over time confirms prediction ANR4.

*Geue* et al. (2014) [44], Scotland, rationing

The authors dispose of a panel covering some 141,000 individuals 45 years and older from 1991 to 2001. They perform survivor analysis using a Gompertz distribution in order to be able to estimate TTD values for survivors, without systematically distinguishing between females and males. While finding support of the RHH, they emphasize the (negative) interaction between TTD and age which becomes more marked with increasing age in the regression designed to explain the occurrence of positive HCE. However, these interaction terms are nonsignificant in the regression for cost ratios (the benchmark *cost* = 1.00 being at age = 45–64 and TTD = 20 quarters) with very few exceptions.

Predictions AR1a and AR2a (which refer to young to medium age) cannot be tested because when the authors vary TTD as well as age (as in their Table 6), they start at age 65–69. This does not apply to AR1b, which however fails to be confirmed because a movement from the age group 75–79 years up to 90+ years combined with one from 19 ≥ TTD ≤ 15 to 14 ≥ TTD ≤ 1 is not associated with increasing cost ratios. As to AR2b, it is vindicated since regardless of TTD, the age profile of HCE stays flat (the cost ratios do not differ from 1.00 and are even lower than 1.00 in three cases).

Finally, the authors do not report the change in the age profile of HCE over time, so predictions AR3 and AR4 cannot be tested either. 

*Gregersen* (2014) [32], Norway, some rationing

The database of this study is described in Section 3.1.2 above (see also Table A1 of Appendix B).

Prediction AR1a is contradicted because in the author’s Figure 1, fitted hospital HCE increases among young adults. AR1b is confirmed in that HCE increases between ages 70 and 85 for both genders. As to AR2a, it is contradicted. In the author’s Figure 2, HCE in the last year before death is shown as a function of age; between the ages 20–24 and 50 (women) and 20–24 and 55–59 (men), it mostly increases rather than decreases. However, AR2b is confirmed by the author’s Table 3 which shows that the age profiles of HCE become flatter beyond the 75–79 age bracket. 

According to [47], life expectancy of Norwegian women increased from 81.3 years in 2000 to 83.1 years in 2010, suggesting that they benefitted from new medical technology, boosting their willingness to pay (WTP). In the author’s Figure 1, there is a clear steepening of the age profile of HCE for between 1998–2003 and 2004–2009 between ages 40 and 83 among women (60 and 87 for men, respectively), but with the shift increasing with age so partially in line with prediction AR3. 

Prediction AR4 cannot be tested because the shift in HCE over time is not displayed with TTD controlled for. 

*Hashimoto* et al. (2010) [28], Japan, no rationing

The database pertaining to this study is described in Section 3.1.2. 

The authors’ Table 2 can once again be exploited for testing. According to prediction ANR1, HCE increases with age at a rate that depends positively on current HCE if TTD is not held constant. With age groups 65–74, 75–84, and 85+, two genders, and four components of HCE, there are 16 testing possibilities among survivors. In 14 instances, HCE increases with age; moreover, the amount of increase depends positively on the amount of HCE in the next-lower age group, constituting strong confirmation of ANR1 (the two exceptions concern expenses on outpatient care).

Among decedents, TTD does not vary more than three years so is held constant at least to an approximation. Here, ANR2 predicts a flat age profile of HCE. It is fully confirmed in the eight comparisons involving outpatient and inpatient expenditure (HCE even tends to decrease with age). However, it is contradicted in the other eight comparisons involving homecare and institutional care, resulting in but partial confirmation. As Hashimoto et al. note, the RHH was originally formulated in the context of medical interventions that held the promise of restoring health rather than long-term care (see Figure 1 again); therefore, these contradictions are not surprising. 

Finally, ANR3 and ANR4 cannot be tested because the dates are of the cross-section type.

*Hazra* et al. (2017) [45], UK, rationing)

The authors have access to data covering some 98,000 individuals aged 80+ over the years 2010 to 2014. They estimate third-degree polynomials of *Age* in their regression to find that in the last year of life (i.e., with TTD held constant), predicted HCE does not increase with age, arguably supporting the RHH (in their Table 2, it even decreases with age at very high ages). Although the authors state as their objective to test the RHH, they abstain from issuing a verdict, emphasizing the importance of comorbidities instead. 

Predictions AR1a and AR2a cannot be tested because the database does not contain patients at young to medium age. However, AR1b is confirmed by the left-hand panel of the authors’ Figure 2, which shows an increase in HCE up to age 100 among women and 97 among men, respectively. Prediction AR2b also receives empirical support since the in the last year before death, the right-hand panel of the authors’ Figure 2 exhibits an age profile of men’s HCE that becomes flatter with increasing age (and even has negative slope for women, as noted above). 

Since prediction AR3 refers to the general population, it cannot be tested. The same is true of AR4 since changes over time are not documented. 

*Karlsson* et al. (2016) [29], German private health insurer, no rationing 

The database for this study is described in Section 3.1.2 above.

Prediction ANR1 is supported by the authors’ Figure 5 which displays a roughly constant rate of increase in HCE up to age 65, accelerating afterwards where HCE is highest (in the 65+ category according to the authors’ Table 5). ANR2 cannot be tested because when holding TTD constant, the authors do not distinguish between age classes.

As to ANR3, it is confirmed indirectly using the authors’ Figure 9 which shows the cdfs pertaining to 2011 and 2015. In 2011, 50 percent of HCE are reached at an estimated value of USD 6920, while in 2015, they are reached at USD 6240 already. Since according to the authors’ Table 5, the highest HCE are exhibited in the 25–64 age group across all quintiles and for both genders, this shift in the cdf reflects a steepening of the age profile of HCE. Finally, ANR4 cannot be tested because the authors do not display a cdf with TTD controlled for.

*Karlsson* et al. (2020) [43], German private health insurer, no rationing

The database is the same as the one described in Section 3.1.2; however, the authors provide more detailed analysis of the effect of age on HCE. Finding a positive age gradient in HCE also when controlling for TTD, they conclude that the RHH is rejected.

Yet their Table 3 provides support for ANR1 in that HCE (with the only exception of the 65–69 age bracket) increases with patient age, with the rate of increase consistently rising with age (and hence HCE). However, the same Table 3 contradicts ANR2 because with TTD controlled for, as HCE again increases with age (albeit at a reduced rate). 

Predictions ANR3 and ANR4 cannot be tested because in their extrapolations, the authors do not report age gradients of HCE.

*Kolodziejczyk* (2020) [46], Denmark, some rationing

The author has a database covering 2371 twins aged 70 or more in 1999, 60 percent of whom were deceased by the end of 2010. The age at death of the co-twins as well as their mother’s serve as instruments for endogenizing time to death (TTD). On the basis of a range of estimations using annual HCE data from 1999 to 2006, the author concludes that earlier contributions likely overestimated the impact of RLE on HCE without issuing a verdict concerning the RHH. However, TTD has a significantly negative coefficient, while *Age* lacks significance in four of age regression results, with the negative coefficient of Age squared rendering the marginal effect of age negative rather than positive. To illustrate, the highest positive coefficient of Age (=0.2081, 2SLS estimation, author’s Table 4) is used. When combined with the −0.1442 pertaining to *Age squared* and evaluated at the mean of 0.744 for *Age*/100, the marginal effect of *Age* on *logHCE* amounts to 0.2081-2˗2∙0.1442∙0.744 = −0.0065 < 0. Therefore, this study can be said to broadly confirm the RHH. 

Predictions AR1a and AR1b cannot be tested because the author controls for TTD throughout. As to AR2a, it is contradicted by the author’s Table 4. Regardless of estimator, the marginal overall effect of *Age* is positive rather than negative at age 40 (say) because the negative coefficient of *Age squared* is too small. For instance, the 2SLS estimate amounts to ∂logHCE/∂Age=0.2081−2⋅0.1442⋅0.4=0.093>0. However, AR2b is confirmed because at age 85, the estimate shrinks to ∂logHCE/∂Age=0.2081 −2⋅0.1442⋅0.85=0.2081−2⋅0.1442⋅0.85=−0.037<0. Predictions AR3 and AR4 cannot be tested since the author does not document changes over time.

*Lorenz* et al. (2020) [34], Germany, rationing

The database of this study is described in Section 3.1.2 above.

Panel (b) of the authors’ Figure 2 provides partial support of prediction AR1a in that women’s HCE decreases between the ages 30 and 42 (i.e., beyond childbearing age at least in part) and men’s up to age 20. AR1b is fully supported by the same source because women’s HCE increases between ages 65 and 78 (men’s, between 65 and 77). 

Prediction AR2a is also confirmed by panels (a) and (b) of the authors’ Figure 3, where TTD is held constant. Women’s HCE falls between ages 30 and 65 (with three short-lived exceptions), which is also true of men’s HCE (again with exceptions, the major one being between ages 25 and 60 for those one year away from death). In return, the rate of decrease tapers off with patient age, as predicted. According to the same source, AR2b is confirmed as well since beyond age 80 for both genders, HCE three years, two years, and one year before death hardly differs, indicating a flat age profile of HCE (with a spike in the last year before death). 

Prediction AR3 is confirmed in part only. According to panel (a) of the authors’ Figure 5, among women only girls and the age bracket 25–42 exhibit HCE that grow faster than the general trend; among men, this is true only for boys. Panel (b) of the same figure supports AR4, showing that in their last year of life, both women’s and men’s HCE converge to the general trend after age 70, with the rate of convergence faster among men. Finally, among women at least, the rate of convergence decreases with age, as predicted. 

*Moorin* et al. (2012) [30], Western Australia, no rationing

The database of this study is described in Section 3.1.2 above.

When reporting age profiles of HCE, the authors hold RLE constant, obviating a test of prediction ANR1. As to ANR2, it is partially confirmed. The authors’ Figure 1 displays the three components of fitted HCE (a: primary care, b: specialist services, and c: diagnostic and therapeutic service) during the last year of life. With three eras and the two genders distinguished, there are 18 age profiles of HCE. Component (a) displays an increase with age for both genders across the three eras distinguished by the authors, accounting for six cases. Components (b) and (c) show four weakly positive and eight negative relationships (whether or not their slopes are significantly different from zero is unclear because no standard errors of regression coefficients are given). 

Prediction ANR3 is partially confirmed, too. The authors’ Figure 1 distinguishes three eras of four years length, 1990–1994, 1995–1999, and 2000–2004. Australian women saw their RLE at age 65 from 16 years in 1960–1962 to 22 years by 2011–2013 [38] presumably also because they had access to improved medical technology, boosting their WTP. In the 10 (out of 18) instances where fitted HCE increases with age, the age profile never steepens consistently across the three eras. The age profile of men’s HCE does become steeper across all three components from 1990–1994 to 1995–1999 but becomes flatter afterwards. The profile of women’s component (a) of HCE steepens from 1995–1999 to 2000–2004. As to ANR4, support is again but partial because in the authors’ Figure 1, the age profiles of HCE among those aged 85+ (many of whom are close to death) become flatter from 1995–1999 to 2000–2004 in category (a), more negative from 1995–1999 to 2000–2004 in category (b), and consistently more negative in category (c).

**Conclusion** **3.** In the 35 instances where the published results are sufficiently detailed to permit testing with regard to the age profile of HCE, in the case of no rationing seven out of twelve are in full and four in partial support of the RHH; in the case of rationing, eleven out of 23 are in full and nine in partial support. As to the three cases where the presence (absence, respectively) of rationing makes a difference, both AR1a and ANR1 are confirmed five out of eleven times but both AR2a and ANR2 as well as AR2b andANR2, zero times.

## 4. Conclusions

The objectives of this contribution are to provide some theoretical underpinning to the ‘red herring’ hypothesis (RHH), to derive two new sets of predictions amenable to empirical testing, and to pit them against available evidence. First, the RHH is extended to apply to a rationed settings as well by modeling healthcare expenditure (HCE) as the resultant of patients’ effort to obtain the desired amount of HCE and the physician’s rationing effort. Next, for deriving testable predictions regarding gender differences in HCE, women’s higher remaining life expectancy RLE (longer time to death, respectively) is used. A complication is that their willingness to pay for health care is also higher than men’s, while their lower income does not matter since the evidence comes from countries and setting with comprehensive health insurance coverage. A second set of predictions refers to the age profile of HCE. Here, an important distinction is whether or not time to death (RLE, respectively) is held constant. 

Among the 15 studies identified in the existing literature that permit at least two tests of the RHH, a majority of 80 percent are in support of the RHH, comprising also six that come from countries and settings subject to at least some rationing. Among the 28 opportunities of testing for the predicted gender-specific differences in HCE (in 16 cases, no test was possible), eleven relate to a non-rationing background and 17, to a rationing one. In the non-rationed category, the RHH is fully confirmed seven times (twice, partially); there are two rejections. Among the 17 opportunities from a rationing background, 12 provide full (one, partial) support of the RHH. Overall, there are seven contradictions.

Turning to of the age profile of HCE predicted by the RHH, one can identify 35 testing opportunities (in no fewer than 20 cases, no test was possible). The twelve opportunities from a non-rationed setting yield seven full (four partial) confirmations of the RHH; the 23 from rationed countries and settings, eleven full (another nine, partial) confirmations. Overall, there are four contradictions.

Therefore, the new, rather fine tests developed in this paper result in a somewhat weaker support of the RHH than the 80 percent in the retained published literature. In particular, out of a total of 63 (=28 + 35) testing opportunities, 37 (=7 + 12 + 7 + 11, or 59 percent) result in full confirmation, 16 (=2 + 1 + 4 + 9, 25 percent) in partial confirmation, and ten (=2 + 4 + 1 + 3, 16 percent) in a contradiction. Of the 37 full confirmations, 14 (=7 + 7, 38 percent) come from non-rationed countries and settings, while 23 (=12 + 11, 62 percent) come from rationed ones. Therefore, although the RHH was originally developed in a non-rationed context, it receives a greater degree of confirmation in rationed ones. 

This surprising result suggests that regardless of the presence or absence of rationing, it would be appropriate for policy-makers concerned by surging HCE to be open about the fact that the issue is not so much the aging of population but rather whether to grant general access to costly new medical technology. However, it may also reflect one of the limitations of this work. In particular, in the absence of rationing there may still be physician influence originating from the patient-physician interaction, which is neglected here. Additionally, the rationing effort by physicians is not derived from a behavioral model; rather it is assumed to be governed by effectiveness and equity considerations in a very simple way. Moreover, all predictions are derived from the ‘red herring’ hypothesis without specification of a competing alternative. Another limitation is that studies which might have provided additional opportunities for testing may have been overlooked. More specifically, the evidence comes almost exclusively from industrialized countries, where women not only have a higher remaining life expectancy than men but also can express their higher demand for healthcare services (reflected by their high maximum willingness to pay a). This fact may limit the applicability of the RHH beyond industrial countries. Finally, the interpretation of the results of the retained studies might be biased in favor of the RHH (or simply erroneous). However, in spite of these limitations, it seems worthwhile to continue examining gender differences in HCE as well as age profiles of HCE in the light of the ‘red herring’ hypothesis.

## Data Availability

The data supporting this study are created by the author. The data used in the works cited would have to be requested from the authors of these works.

## Appendix A. Derivation of the Nash Equilibrium

From the first-order condition (FOC) with respect to $e^{P}$ for an interior solution [Equation (3) of the text], one obtains the patient’s comparative-static equation determining the optimal response to a shock $de^{R}>0$ as

(A1) $$\left\{\frac{\partial^{2}u^{P}}{\partial x^{P2}}\frac{\partial x^{P}}{\partial e^{P}}+\frac{\partial u^{P}}{\partial x^{D}}\frac{\partial^{2}x^{P}}{\partial e^{P2}}\right\}de^{P}+\left\{\frac{\partial^{2}u^{P}}{\partial x^{P2}}\frac{\partial x^{P}}{\partial e^{R}}+\frac{\partial u^{P}}{\partial x^{P}}\frac{\partial^{2}x^{P}}{\partial e^{P}\partial e^{R}}\right\}de^{R}=0\;$$

Assuming that the patient sets effort in an attempt at maximizing utility rather than minimizing disutility (after all, the ultimate target is health), the first term must be negative in view of the second-order condition. This implies that the second term is positive, hence

(A2) $$\frac{de^{P}}{de^{R}}=-\frac{\frac{\partial^{2}u^{P}}{\partial x^{P2}}\frac{\partial x^{P}}{\partial e^{R}}+\frac{\partial u^{P}}{\partial x^{P}}\frac{\partial^{2}x^{P}}{\partial e^{P}\partial e^{R}}}{\frac{\partial^{2}u^{P}}{\partial x^{P2}}\frac{\partial x^{P}}{\partial e^{P}}+\frac{\partial u^{P}}{\partial x^{P}}\frac{\partial^{2}x^{P}}{\partial e^{P2}}}>0$$

is the slope of the patient’s response function. Since the values of this function are not known, the patient’s response function is depicted as a straight line in Figure A1a for simplicity.

For the rationing physician, the comparative-static equation becomes in view of both Equations (6) and (7),

(A3) $$\left\{\frac{\partial^{2}u^{R}}{\partial x^{R2}}\frac{\partial x^{R}}{\partial e^{R}}+\frac{\partial u^{R}}{\partial x^{R}}\frac{\partial^{2}x^{R}}{\partial e^{R2}}\right\}de^{R}+\left\{\frac{\partial^{2}u^{R}}{\partial x^{R2}}\frac{\partial x^{R}}{\partial e^{P}}+\frac{\partial u^{R}}{\partial x^{R}}\frac{\partial^{2}x^{R}}{\partial e^{R}\partial e^{P}}\right\}de^{P}=0\;$$

in full analogy with Equation (A1). Rationing effort arguably gives rise to disutility at the margin, implying that the first term is negative if $\partial u^{R}/\partial x^{R}>0$ and negative also if $\partial u^{R}/\partial x^{R}<0$. Therefore, the second term must be positive to satisfy Equation (A.3), yielding

(A4) $$\frac{de^{R}}{de^{P}}=-\frac{\frac{\partial^{2}u^{R}}{\partial x^{R2}}\frac{\partial x^{R}}{\partial e^{P}}+\frac{\partial u^{R}}{\partial x^{R}}\frac{\partial^{2}x^{R}}{\partial e^{R}\partial e^{P}}}{\frac{\partial^{2}u^{R}}{\partial x^{R2}}\frac{\partial x^{R}}{\partial e^{R}}+\frac{\partial u^{R}}{\partial x^{R}}\frac{\partial^{2}x^{R}}{\partial e^{R2}}}>0.$$

The physician’s response function is again depicted as a straight line in Figure A1a for simplicity.

It becomes evident from panel (a) that a Nash equilibrium (NE) with positive effort levels is likely to exist. For zero effort levels, the physician’s response function would have to run higher than the patient’s for all values of patient effort $e^{P}$—a rather restrictive requirement (see dashed lines in panel (a)). As drawn, the Nash equilibrium is stable because an off-equilibrium point such as P initiates an adjustment process that converges at NE (on the properties of the Nash equilibrium and the stability condition, see e.g., Mas-Collel, Whinston and Green (1995, [48], chs. 8.D and and 12.G).

Figure A1b exhibits the projection of effort levels *e^P^*^*^ and *e^R^*^*^ associated with the NE into HCE-space. Three outcomes can be distinguished:

$$•\;HCE^{P}\left[e^{P*}\right]=HCE^{R}\left[e^{R*}\right]$$

The patient’s effort at obtaining care results in the same amount of HCE as the physician’s rationing effort. Treatment takes place, but observed HCE reflects the intended amounts of both the patient and the physician, as in Appendix D and Appendix E in the presence of rationing.

$$•\;HCE^{P}\left[e^{P*}\right]>HCE^{R}\left[e^{R*}\right]$$

The patient’s effort at obtaining care results in an amount of HCE that exceeds the amount associated with by the physician’s rationing effort. Whether or not to accept this outcome is the patient’s decision. In the case of acceptance, treatment takes place but observed HCE reflects the amount intended by the physician in principle. In the case of non-acceptance, the patient may search for an accommodating provider, resulting in the first outcome; otherwise, HCE is zero (almost never recorded in the papers discussed in Section 3.1 and Section 3.2). In sum, this outcome is indistinguishable from the first one.

$$•\;HCE^{P}\left[e^{P*}\right]<HCE^{R}\left[e^{R*}\right].$$

The patient’s effort at obtaining care results in an amount of HCE that falls short of the amount offered by the physician. In a rationed context, this is a very unlikely outcome; in a non-rationed one, this case would reflect supplier-induced demand if the physician prevails.

Observed amounts of HCE in a rationed setting will be therefore analyzed assuming the first outcome, permitting application of the pertinent predictions of Appendix D and Appendix E.

## Appendix B

**Table A1:** Retained sources of the evidence concerning the ‘red herring’ hypothesis (RHH).

Authors	Type of Data	Country	Rationing?	In Support of RHH?
1. Atella and Conti (2014) [49]	Fixed panel 2006–2009, three categories of outpatient HCE	Italy	**No** (Donatini et al. (2001), p. 101 [50])	**Yes** ^1^
2. Bjørner and Arnberg (2012) [41]	Panel 2000–2009, inpatient and outpatient HCE	Denmark	**Some** (Vallgårda et al. (2001), pp. 74–75 [51]; Cylus and Papanicolas (2015) [52])	**Yes** ^2^
3. Breyer et al. (2015) [27]	Pseudo-panel 1997–2009, 2340 age and sex groups	Germany	**Yes** (Nadolski (2002) [53]; Thielscher et al. (2012) [54]; Cylus and Papanicolas (2015) [52])	**No**
4. Costa-Font and Vilaplana-Prieto (2020) [31]	Pseudo-panel, waves 1, 2 and 4–7 of SHARE, 288,600 observations	17 countries	**Yes** (the majority of individuals sampled are subject to rationing)	**No** ^1^
5. De Nardi et al. (2016) [42]	Panel 1996–2010, 67,000 Medicare enrollees	United States	**No** (Nelson (2011) [55])	**Yes**
6. Gregersen (2014) [32]	Pseudo-panel, entire population of 5 mn, 1998–2009	Norway	**Some** (Cylus and Papanicolas (2015) [52])	**Yes**
7. Hashimoto et al. (2010) [28]	Panel 2000–2004 aged 65+, 354,500 survivors, 5099 decedents	Japan	**No** (personal communication from H. Hashimoto; Gaille, 2019 [56])	**Yes** ^3^
8. Hazra et al. (2017) [45]	Panel 2010–2014, 98,000 aged 80+	United Kingdom	**Yes** (Light (1997) [57]; Cylus and Papanicolas (2015) [52])	**Yes** ^3^
9. Howdon and Rice (2018) [5]	Panel 2005–2012, 40,000 suvivors and decedents each	United Kingdom	**Yes** (Light, 1997 [57]; Cylus and Papanicolas, 2015 [52])	**Yes**
10. Karlsson et al. (2020) [43]	Pseudo-panel, members of private health insurer, 2005–2011, 8.7 mn observations	Germany	**No** ^4^	**No**
11. Karlsson et al. (2016) [29]	Pseudo-panel, members of private health insurer, 2005–2011, 8.7 mn observations	Germany	**No** ^4^	**Yes**
12. Kolodziejczyk (2020) [46]	Panel of twins, 1999–2010, aged 70+	Denmark	**Some** (Vallgårda et al. (2001), pp. 74–75 [51]; Cylus and Papanicolas (2015 [52])	**Yes**
13. Moorin et al. (2012) [30]	All deaths 1990–2004, three categories of outpatient HCE	Western Australia	**Not** until 2010 (Baume (1998) [58]; O’Connor (2010) [59])	**Yes** ^5^
14. Seshamani and Gray (2004) [39]	Inpatient HCE	United Kingdom	**Yes** (Light (1997) [57]; Cylus and Papanicolas (2015) [52])	**Yes**
15. Seshamani and Gray (2004) [35]	Inpatient HCE	United Kingdom	**Yes** (Light (1997) [57]; Cylus and Papanicolas (2015) [52])	**Yes**
16. Wei and Zhou (2020) [36]	China Health and Retirement Longitudinal Study 2011 & ’13	China	**Yes** (Fang, 2020) [60]	**Yes**

^1^ Age is a significant independent predictor but dominated by TTD. ^2^ Cohort effects and increases in life expectancy important for forecasting HCE. ^3^ Time to death significant but exhibits decreasing effect at very high ages. ^4^ Time to death significant but exhibits decreasing effect at very high ages. ^5^ The data refer to privately insured individuals. ^6^ Drop in expenditure on specialist services in the last month before death as one contradiction.

## Appendix C. Deriving Estimates of the Parameter *b* in Equation (1)

The study by [1] refers to a setting without rationing. The dependent variable is the log of quarterly real HCE measured from 1993 to 1992; it increased from CHF (Swiss francs) 408 to 1098 (see text below their Figure 2). In their Table 3, the last quarter prior to death (Q1) has a coefficient of 1.849, compared to 0 in quarter no. 20 (Q20, the benchmark), with age and gender held constant. Using the average HCE of CHF 753 over the observation period, the transition from Q20 to Q1 is therefore associated with an increase in HCE by CHF 2145 (=2.849 ∙ 753) over 5 years (most of the evidence discussed in Section 3.1 and Section 3.2 refers to annual data). Now according to Equation (1) of the text, this increase corresponds to $2b\left(a-bT\right)$, with a−bT equal to HCE. Using the average of CHF 753 once again, one obtains

(A5) 2b⋅753=2145→b=1.424 per 20 quarters, →b=0.285 per year, 

a value clearly below one for the general population.

However, the value of *b* may well be higher in the year prior to death. The coefficient pertaining to Q5 is 0.459, leading to an estimated HCE of CHF 1099 (=1.459 ∙ 753) in excess of the benchmark. The transition from Q5 to Q1 is therefore associated with an increase in HCE amounting to CHF 713 (=1812–1099) over four quarters. In analogy to Equation (A5), one obtains

(A6) 2b⋅713=1812→b=1.271 per 4 quarters, →b=1.271 per year, 

a value clearly above one in the year prior to death (at the latest, quite possibly already earlier), years which predominantly occur at high to very high age.

## Appendix D. W-Predictions Concerning the Effect of Women’s Higher RLE and Higher WTP on HCE under the ‘Red Herring’ Hypothesis (RHH)

As noted in Section 1, women not only have higher RLE but also higher WTP *ceteris paribus* (this clause is satisfied in the studies analyzed, which come from countries and settings with comprehensive health insurance coverage).

### Appendix D.1. Absence of Rationing

In the absence of rationing, one can use Equation (1) to obtain the combined effect of women’s higher RLE (denoted by ∂T>0) and WTP. Strictly speaking, it would be preferable to add two elasticities in Equation (A7) below to make the two components commensurable; however, this would complicate the analysis considerably. An alternative is to think of a unit change as a variation by one standard deviation. With $\Sigma^{NR}$ denoting this combined effect, one has

(A7) $$\begin{array}{ll} \Sigma^{NR} & =\frac{\partial x^{P}}{\partial T}+\frac{\partial x^{P}}{\partial a}=\frac{\partial }{\partial T}{\left(a-bT\right)}^{2}+\frac{\partial }{\partial a}{\left(a-bT\right)}^{2}\\ & =-2b\left(a-bT\right)+\;2\left(a-bT\right)=2\left(1-b\right)\left(a-bT\right)\\ & \;>\;0\;\mathrm{if}\;b<1\;(\mathrm{WNR}1;\;\mathrm{applies}\;\mathrm{to}\;\mathrm{the}\;\mathrm{general}\;\mathrm{population}\;\\ & \;(\mathrm{see}\;\mathrm{Appendix}\;C),\;\mathrm{value}\;\mathrm{depending}\;\mathrm{positively}\;\mathrm{on}\;\mathrm{current}\;\mathrm{HCE});\;\\ & \;<\;0\;\mathrm{if}\;b>1\;(\mathrm{WNR}2;\;\mathrm{applies}\;\mathrm{to}\;\mathrm{the}\;\mathrm{last}\;\mathrm{year}\;\mathrm{before}\;\mathrm{death}\;\mathrm{at}\;\mathrm{the}\;\mathrm{latest}\;\\ & \;(\mathrm{see}\;\mathrm{Appendix}\;C),\;\mathrm{absolute}\;\mathrm{value}\;\mathrm{depending}\;\mathrm{positively}\;\mathrm{on}\;\mathrm{current}\;\mathrm{HCE}). \end{array}$$

since a−bT>0 to ensure positive HCE. In Appendix C, estimates of b are derived; with b≈0.3, it is clearly below one for the general population. However, with b≈1.3, it is above one in the last year before death. Thus under the RHH, with b<1 women’s HCE is higher than men’s except in the year before death at the latest when b>1, with the difference depending positively on current HCE. This is entered as predictions WNR1 and WNR2 in Table 1.

As to the *effect of a change in RLE* (proximity to death, respectively), one has from Equation (A7)

(A8) $$\begin{array}{l} \frac{\partial \Sigma^{NR}}{\partial T}=2\left(1-b\right)\left(-b\right)=-2b\left(1-b\right)\\ \;<\;0\;\mathrm{if}\;b<1\;(\mathrm{WNR}3;\;\mathrm{applies}\;\mathrm{to}\;\mathrm{the}\;\mathrm{general}\;\mathrm{population}\;(\mathrm{see}\;\mathrm{Appendix}\;C));\\ \;>\;0\;\mathrm{if}\;b>1\;(\mathrm{WNR}4;\;\mathrm{applies}\;\mathrm{to}\;\mathrm{the}\;\mathrm{year}\;\mathrm{before}\;\mathrm{death}\;\mathrm{at}\;\mathrm{the}\;\mathrm{latest}\;\\ \;(\mathrm{see}\;\mathrm{Appendix}\;C)).\; \end{array}$$

Except shortly before death, women’s HCE is predicted to decrease more slowly than men’s with increasing RLE (recall the positive value of Equation (A7)); conversely, it *increases* more slowly at a constant rate than men’s with closeness to death. Conversely, shortly before death women’s HCE increases faster with closeness to death (now the value of Equation (A7) is negative) than men’s, at a constant rate in both cases.

Finally, the *increase in maximum WTP over time due to new in medical technology* always goes along with an increase in RLE, as evidenced in Section 3.1.2. Therefore, one obtains from Equation (A8),

(A9) $$\frac{\partial^{2}\Sigma^{NR}}{\partial a\partial T}=0\;(\mathrm{WNR}5),\;$$

implying that in the absence of rationing, any difference between women’s and men’s HCE remains constant over time.

### Appendix D.2. Presence of Rationing

In a rationing context, the Nash equilibrium implies the equality $x^{P}\left(e^{P}*\right)=$$x^{R}\left(e^{R}*\right):=x$, with $e^{P}*$ and $e^{R}*$ denoting optimum effort levels. Therefore, $x={\left(a-bT\right)}^{2}=f+g\left(T/A\right)$. Analyzing $x^{2}=x\cdot x$ by imposing this equality and multiplying Equation (1) by Equation (4) turns out to be much simpler than analyzing x itself. Note that the partial derivatives of $x^{2}$ with respect to A and T have the same sign as those of x while there is no need to determine the location of the Nash equilibrium. However, this implies that no derivatives with respect to x can be formed and evaluated. One therefore obtains

(A10) x2=a−bT2f+gT/A, with ∂x∂x2∂x2∂A=12xa−bT2−gT/A2<0 and∂x∂x2∂x2∂T=12xa−bT−2bf+gT/A+a−bT22g1/A0<>.

Therefore, the RHH predicts that HCE decreases unambiguously with age due to the influence of the rationing physician, while age per se does not matter to the patient. With regard to remaining life expectancy T, there are two opposing forces. On the one hand, patients are less interested in HCE when their RLE is higher (giving rise to the negative first term in the large bracket); on the other hand, the physician’s rationing effort decreases with higher RLE (giving rise to the positive second term).

For the combined effect of women’s higher RLE and WTP, one obtains

(A11) $$\begin{array}{cc} \Sigma^{R} & =\frac{\partial x}{\partial T}+\frac{\partial x}{\partial a}=\frac{\partial x}{\partial x^{2}}\left\{\frac{\partial x^{2}}{\partial T}+\frac{\partial x^{2}}{\partial a}\right\}\\ \; & \;=\frac{1}{2x}\left\{\begin{array}{c} \left(a-bT\right)\left(-2b\right)\left\{f+g\left(T^{2}/A\right)\right\}\\ +{\left(a-bT\right)}^{2}\left(2gT/A\right)+2\left(a-bT\right)\left\{f+g\left(T^{2}/A\right)\right\} \end{array}\right\}\\ & \;=\frac{1}{x}\left\{\begin{array}{c} -b\left(a-bT\right)\left\{f+g\left(T^{2}/A\right)\right\}\\ +g{\left(a-bT\right)}^{2}\left(T/A\right)+\left(a-bT\right)\left\{f+g\left(T^{2}/A\right)\right\} \end{array}\right\}\\ & \;\approx -b\left\{f+g\left(T^{2}/A\right)\right\}+g\left(a-bT\right)\left(T/A\right)+\left\{f+g\left(T^{2}/A\right)\right\}\\ & \;\approx \left(1-b\right)\left\{f+g\left(T^{2}/A\right)\right\}+g\left(a-bT\right)\left(T/A\right)\;\\ & \;>0\;(\mathrm{WR}1;\;\mathrm{applies}\;\mathrm{to}\;\mathrm{the}\;\mathrm{general}\;\mathrm{population}\;\mathrm{because}\;b<1\;(\mathrm{see}\;\mathrm{Appendix}\;C),\\ & \;\mathrm{with}\;\mathrm{the}\;\mathrm{difference}\;\mathrm{in}\;\mathrm{favor}\;\mathrm{of}\;\mathrm{women}\;\mathrm{depending}\;\mathrm{negatively}\;\mathrm{on}\;\mathrm{patient}\;\mathrm{age});\\ & \;<0\;(\mathrm{WR}2;\;\mathrm{applies}\;\mathrm{to}\;\mathrm{the}\;\mathrm{last}\;\mathrm{year}\;\mathrm{before}\;\mathrm{death}\;\mathrm{at}\;\mathrm{the}\;\mathrm{latest}\;\mathrm{because}\;b>1\;\\ & \;(\mathrm{see}\;\mathrm{Appendix}\;C),\;\mathrm{with}\;\mathrm{the}\;\mathrm{difference}\;\mathrm{to}\;\mathrm{the}\;\mathrm{detriment}\;\mathrm{of}\;\mathrm{women}\;\mathrm{depending}\\ & \;\mathrm{negatively}\;\mathrm{on}\;\mathrm{patient}\;\mathrm{age}).\; \end{array}$$

The approximation holds because observed HCE cannot differ too much from the desired amount $\left(a-bT\right)$ lest the tension between patient and physician becomes excessive, in which case there is no treatment hence HCE = 0. Thus, except shortly before death women are predicted to exhibit higher HCE than men in a rationed setting. These are WR1 and WR2 in Table 1.

As to *the rate of change of HCE as a function of remaining life expectancy* (holding the gender difference in RLE constant), one obtains from Equation (A11)

(A12) $$\begin{array}{cc} \frac{\partial \Sigma^{R}}{\partial T} & \approx -bg\left(1/A\right)-bg\left(T/A\right)+g\left(a-bT\right)\left(1/A\right)\\ \; & \;+f+gT\left(T/A\right)+T\left\{g\left(T/A\right)+gT\left(1/A\right)\right\}\\ \; & \;\approx \left\{-bg+g\left(a-bT\right)+gT^{2}\right\}\left(1/A\right)+\left\{-bg+2gT\right\}\left(T/A\right)+f\\ \; & \;\approx f+g\left\{-b+\left(a-bT\right)+T^{2}\right\}\left(1/A\right)+g\left\{-b+2T\right\}\left(T/A\right)\\ \; & \;>0\;(\mathrm{WR}3;\;\mathrm{applies}\;\mathrm{to}\;\mathrm{the}\;\mathrm{general}\;\mathrm{population},\;\mathrm{with}\;\mathrm{the}\;\mathrm{difference}\;\\ \; & \;\mathrm{depending}\;\mathrm{negatively}\;\mathrm{on}\;\mathrm{patient}\;\mathrm{age}).\\ \; & \; \end{array}$$

since both brackets are positive. Therefore, given rationing and in the general population women exhibit HCE that *decreases* at a lower rate than men’s with increasing RLE (*increases* at a lower rate than men’s with proximity death, respectively; see Equation (A11) again).

In the year before death at the latest, one has with T→0

(A13) $$\begin{array}{l} \frac{\partial \Sigma^{R}}{\partial T}\approx f+g\left(a-b\right)\left(1/A\right)\;\\ >0\;(\mathrm{WR}4;\;\mathrm{applies}\;\mathrm{to}\;\mathrm{the}\;\mathrm{last}\;\mathrm{year}\;\mathrm{before}\;\mathrm{death}\;\mathrm{at}\;\mathrm{the}\;\mathrm{latest},\;\mathrm{with}\;\mathrm{the}\;\\ \;\mathrm{difference}\;\mathrm{depending}\;\mathrm{negatively}\;\mathrm{on}\;\mathrm{patient}\;\mathrm{age}).\; \end{array}$$

Thus, shortly before death women also exhibit HCE that decreases at a lower rate with increasing RLE [recall that Equation (A11) has a negative value in this case] thus increases at a lower rate than men’s with closeness to death. Yet the difference is small compared to that of Equation (A12), where the first bracket now shrinks in value while the second goes to zero.

Finally, the *increase in both maximum WTP and RLE over time due to new medical technology* has also an effect on the gender difference of HCE. From Equation (A13), one obtains

(A14) $$\begin{array}{cc} \frac{\partial^{2}\Sigma^{R}}{\partial a\partial T} & \approx g\left(1/A\right)\\ & >\;0\;(\mathrm{WR}5;\;\mathrm{any}\;\mathrm{gender}\;\mathrm{difference}\;\mathrm{in}\;\mathrm{HCE}\;\mathrm{approaches}\;\mathrm{zero}\\ & \;\;\;\;\mathrm{among}\;\mathrm{the}\;\mathrm{very}\;\mathrm{old}\;\sin\mathrm{ce}\;1/A\to 0). \end{array}$$

Therefore, any difference between women’s and men’s HCE is predicted to decrease over time, with full convergence at high age.

## Appendix E. A-Predictions Concerning the Age Profile of HCE under the ‘Red Herring’ Hypothesis (RHH)

### Appendix E.1. Absence of Rationing

#### Appendix E.1.1. Influence of Age on HCE with Remaining Life Expectancy (Time to Death, Respectively) Not Held Constant

In many published studies, the age profile of HCE is reported without controlling for remaining life expectancy RLE (time to death, respectively). For deriving predictions, one can use the fact that RLE predominantly correlates negatively with age in observed data. Examining the case of perfect negative correlation with dA=−dT is sufficient for the present purpose.

Therefore, in the absence of rationing, one obtains from Equation (1) of the text

(A15) $$\begin{array}{l} \partial x^{P}/\partial A=-\partial x^{P}/\partial T=2b\left(a-bT\right)\\ >\;0\;(\mathrm{ANR}1;\;\mathrm{with}\;\mathrm{the}\;\mathrm{rate}\;\mathrm{of}\;\mathrm{increase}\;\mathrm{depending}\;\mathrm{positively}\;\mathrm{on}\;\\ \;\;\mathrm{current}\;\mathrm{HCE}\;(\mathrm{see}\;\mathrm{Table}\;2)).\; \end{array}$$

Therefore, if RLE (*T* respectively) is not held constant, higher age implies a decrease in *T* and hence an increase in HCE according to the RHH.

#### Appendix E.1.2. Effect of Age on HCE with Remaining Life Expectancy T Held Constant

In the absence of rationing, age per se does not matter according to the RHH [see Equation (1)], thus

(A16) $$\partial x^{P}/\partial A=0\;(\mathrm{ANR}2),\;$$

resulting in a flat age profile of HCE.

#### Appendix E.1.3. Steepening of the Age Gradient of HCE over Time?

The issue of ‘steepening’ of the age gradient of HCE over time has not been resolved. While [11,61] have found evidence to this effect, [62] failed to find it, possibly because their data are from a non-rationed setting. Here, new medical technology boosting WTP is considered as the main change that might cause steepening over time.

Therefore, Equations (A5) and (A6) can be used once again, with dT>0 because RLE increases over time,

(A17) $$\begin{array}{l} \Sigma^{NR}=\frac{\partial x^{P}}{\partial T}+\frac{\partial x^{P}}{\partial a}=2\left(1-b\right)\left(a-bT\right)\\ \;>\;0\;\mathrm{if}\;b<1\;(\mathrm{ANR}3;\;\mathrm{applies}\;\mathrm{to}\;\mathrm{the}\;\mathrm{general}\;\mathrm{population}\;(\mathrm{see}\;\mathrm{Appendix}\;C),\;\\ \;\mathrm{with}\;\mathrm{the}\;\mathrm{rate}\;\mathrm{of}\;\mathrm{increase}\;\mathrm{positively}\;\mathrm{depending}\;\mathrm{on}\;\mathrm{current}\;\mathrm{HCE});\\ \;<\;0\;\mathrm{if}\;b>1\;(\mathrm{ANR}4;\;\mathrm{applies}\;\mathrm{to}\;\mathrm{the}\;\mathrm{last}\;\mathrm{year}\;\mathrm{before}\;\mathrm{death}\;\mathrm{at}\;\mathrm{the}\;\mathrm{latest}\\ \;(\mathrm{see}\;\mathrm{Appendix}\;C),\;\mathrm{with}\;\mathrm{the}\;\mathrm{rate}\;\mathrm{of}\;\mathrm{decrease}\;\mathrm{positively}\;\mathrm{depending}\;\\ \;\mathrm{on}\;\mathrm{current}\;\mathrm{HCE}).\; \end{array}$$

In the general population and in the absence of rationing, the RHH predicts a steepening of the age profile of HCE over time at a rate that depends positively on current HCE. Shortly before death at the latest, however, the predicted age profile becomes flatter at a rate that again depends on current HCE.

### Appendix E.2. Presence of Rationing

For ease of reference, Equation (A10) is repeated here,

(A18) x2=a−bT2f+gT2/A, with ∂x∂x2∂x2∂A=12xa−bT2−gT2/A2<0 and∂x∂x2∂x2∂T=12xa−bT−2bf+gT2/A+a−bT22gT/A0<>. 

#### Appendix E.2.1. Influence of Age on HCE with Remaining Life Expectancy Not Held Constant

With increasing age, RLE decreases *ceteris paribus*, inducing a negative correlation between age and RLE. Once again, examining the case of a perfect negative correlation with dT=−dA is sufficient. Given rationing, the pertinent derivative of Equation (A18) thus reads, recalling that $x.=\mathrm{HCE}\approx \left(a-bT\right)$

(A19) $$\begin{array}{cc} \frac{\partial x}{\partial A}+\frac{\partial x}{-\partial T} & =\frac{\partial x}{\partial x^{2}}\left\{\frac{\partial x^{2}}{\partial A}-\frac{\partial x^{2}}{\partial T}\right\}\\ \; & \;=\frac{1}{2x}\left\{-{\left(a-bT\right)}^{2}g\left(T^{2}/A^{2}\right)-\left\{\begin{array}{c} \left(a-bT\right)\left(-2b\right)\left\{f+g\left(T^{2}/A\right)\right\}\\ +{\left(a-bT\right)}^{2}\left\{2g\left(T/A\right)\right\} \end{array}\right\}\right\}\\ & \;\approx -\frac{1}{2}\left\{\left(a-bT\right)\left\{\begin{array}{c} g\left(T^{2}/A^{2}\right)-2b\left\{f+g\left(T^{2}/A\right)\right\}\\ +2g\left(a-bT\right)\left(T/A\right) \end{array}\right\}\right\}\\ & \;\approx -\frac{1}{2}\left(a-bT\right)\left\{g\left(T^{2}/A^{2}\right)\left\{1-2b/A+2\left(a-bT\right)/\left(T/A\right)\right\}-2bf\right\}\\ & \;<0\;(\mathrm{AR}1a;\;\mathrm{this}\;\mathrm{applies}\;\mathrm{to}\;\mathrm{low}\;\mathrm{to}\;\mathrm{medium}\;\mathrm{age}\;\mathrm{because}\;2\left(a-bT\right)\\ & \;\approx 2\mathrm{HCE}>0\;\mathrm{dominates}\;\mathrm{the}\;\mathrm{bracket}\;\mathrm{since}\;bf<\left(a-bT\right));\;\\ & \;0\;(\mathrm{AR}1b;\;\mathrm{this}\;\mathrm{applies}\;\mathrm{to}\;\mathrm{high}\;\mathrm{age}\;\mathrm{because}\;T/A<1\;\mathrm{hence}\;\\ & \;T^{2}/A^{2}={\left(T/A\right)}^{2}\to 0).\;\\ & \; \end{array}$$

Therefore, with time to death not held constant and in the presence of rationing, HCE is predicted to fall with age first. However, at high age, the value of Equation (A19) approaches $\left(a-bT\right)bf>0$, indicating that HCE increases with age.

#### Appendix E.2.2. Influence of Age on HCE with Remaining Life Expectancy Held Constant

In this case, the derivative of Equation (A18) reads

(A20) $$\begin{array}{cc} \frac{\partial x}{\partial A} & =\frac{\partial x}{\partial x^{2}}\left\{\frac{\partial x^{2}}{\partial A}\right\}\\ & =\frac{1}{2x}\left\{-{\left(a-bT\right)}^{2}g\left(T^{2}/A^{2}\right)\right\}\\ & \approx \frac{-g}{2}\left(a-bT\right){\left(T/A\right)}^{2}\\ & <0\;(\mathrm{AR}3\;(\mathrm{applies}\;\mathrm{to}\;\mathrm{the}\;\mathrm{general}\;\mathrm{population},\;\mathrm{with}\;\mathrm{the}\;\mathrm{rate}\;\mathrm{of}\;\mathrm{decrease}\;\\ & \mathrm{depending}\;\mathrm{negatively}\;\mathrm{on}\;\mathrm{patient}\;\mathrm{age})).\; \end{array}$$

Therefore, in the presence of rationing the RHH predicts a fall of HCE with age at a rate that decreases with age, reflecting the influence of the rationing physician

At very high age $\left(T/A\to 0\right)$, Equation (A20) approaches

(A21) $$\frac{\partial x}{\partial A}=0\;(\mathrm{AR}2b),\;$$

indicating that the age profile of HCE becomes flat; time to death relative to age loses its importance.

As to the issue of *steepening of the age profile of HCE over time*, one can use Equation (A18) again, which combines the effects of higher RLE and higher WTP,

(A22) $$\begin{array}{cc} \Sigma^{R} & =\frac{\partial x}{\partial T}+\frac{\partial x}{\partial a}=\frac{\partial x}{\partial x^{2}}\left\{\frac{\partial x^{2}}{\partial T}+\frac{\partial x^{2}}{\partial a}\right\}\\ \; & \;\approx \left(1-b\right)\left\{f+g\left(T^{2}/A\right)\right\}+g\left(a-bT\right)\left(T/A\right)\\ \; & \;0\;(\mathrm{AR}3;\;\mathrm{this}\;\mathrm{applies}\;\mathrm{to}\;\mathrm{the}\;\mathrm{general}\;\mathrm{population}\;\mathrm{because}\;\\ \; & \;b<1\;(\mathrm{see}\;\mathrm{Appendix}\;C),\;\mathrm{with}\;\mathrm{the}\;\mathrm{rate}\;\mathrm{of}\;\mathrm{increase}\;\mathrm{depending}\;\\ \; & \;\mathrm{negatively}\;\mathrm{on}\;\mathrm{patient}\;\mathrm{age});\\ \; & \;0\;(\mathrm{AR}4;\;\mathrm{this}\;\mathrm{applies}\;\mathrm{to}\;\mathrm{the}\;\mathrm{last}\;\mathrm{year}\;\mathrm{before}\;\mathrm{death}\;\mathrm{at}\;\mathrm{the}\;\mathrm{latest}\\ \; & \;\mathrm{because}\;b>1\;(\mathrm{see}\;\mathrm{Appendix}\;C),\;\mathrm{with}\;\mathrm{the}\;\mathrm{rate}\;\mathrm{of}\;\mathrm{decrease}\;\\ \; & \;\mathrm{depending}\;\mathrm{negatively}\;\mathrm{on}\;\mathrm{patient}\;\mathrm{age}).\; \end{array}$$

Thus, in the general population the RHH predicts a ‘steepening’ of the age profile of HCE over time in the presence of rationing. At very high age, however, it predicts an increasingly flat age profile. In both cases, the rate of change depends negatively on age.
